# Limited Metabolomic Overlap between Commensal Bacteria and Marine Sponge Holobionts Revealed by Large Scale Culturing and Mass Spectrometry-Based Metabolomics: An Undergraduate Laboratory Pedagogical Effort at Georgia Tech

**DOI:** 10.3390/md21010053

**Published:** 2023-01-14

**Authors:** Jessica M. Deutsch, Madison O. Green, Priyanka Akavaram, Ashleigh C. Davis, Sarth S. Diskalkar, Isabelle A. Du Plessis, Hannah A. Fallon, Emma M. Grason, Emma G. Kauf, Zoe M. Kim, Jeffrey R. Miller, Abby L. Neal, Tatiana Riera, Sofie-Ellen Stroeva, Jollin Tran, Vivi Tran, Azucena Velgara Coronado, Vanessa Velgara Coronado, Benjamin T. Wall, Chung mo Yang, Ipsita Mohanty, Nadine H. Abrahamse, Christopher J. Freeman, Cole G. Easson, Cara L. Fiore, Alison E. Onstine, Naima Djeddar, Shweta Biliya, Anton V. Bryksin, Neha Garg, Vinayak Agarwal

**Affiliations:** 1 School of Chemistry and Biochemistry, Georgia Institute of Technology, Atlanta, GA 30332, USA; 2 School of Biological Sciences, Georgia Institute of Technology, Atlanta, GA 30332, USA; 3 Skaggs School of Pharmacy and Pharmaceutical Sciences, University of California San Diego, La Jolla, CA 92093, USA; 4 Department of Biology, College of Charleston, Charleston, SC 29424, USA; 5 Department of Biology, Middle Tennessee State University, Murfreesboro, TN 37132, USA; 6 Department of Biology, Appalachian State University, Boone, NC 28608, USA; 7 Parker H. Petit Institute for Bioengineering and Biosciences, Georgia Institute of Technology, Atlanta, GA 30332, USA; 8 Center for Microbial Dynamics and Infection, Georgia Institute of Technology, Atlanta, GA 30332, USA

**Keywords:** sponge, natural products, metabolomics, mass spectrometry, bacteria

## Abstract

Sponges are the richest source of bioactive organic small molecules, referred to as natural products, in the marine environment. It is well established that laboratory culturing-resistant symbiotic bacteria residing within the eukaryotic sponge host matrix often synthesize the natural products that are detected in the sponge tissue extracts. However, the contributions of the culturing-amenable commensal bacteria that are also associated with the sponge host to the overall metabolome of the sponge holobiont are not well defined. In this study, we cultured a large library of bacteria from three marine sponges commonly found in the Florida Keys. Metabolomes of isolated bacterial strains and that of the sponge holobiont were compared using mass spectrometry to reveal minimal metabolomic overlap between commensal bacteria and the sponge hosts. We also find that the phylogenetic overlap between cultured commensal bacteria and that of the sponge microbiome is minimal. Despite these observations, the commensal bacteria were found to be a rich resource for novel natural product discovery. Mass spectrometry-based metabolomics provided structural insights into these cryptic natural products. Pedagogic innovation in the form of laboratory curricula development is described which provided undergraduate students with hands-on instruction in microbiology and natural product discovery using metabolomic data mining strategies.

## 1. Introduction

Marine sponges are surface-tethered invertebrate metazoans that derive nutrition via filter feeding. The filter feeding activity of sponges is proposed to be responsible for nutrient circulation in benthic ecosystems [1,2,3]. Due to their sessile nature, sponges have developed elaborate chemical defenses in the form of small organic molecules—colloquially referred to as natural products—to prevent predation and herbivory [4,5,6]. Sponges are one of the richest sources of natural products in the marine environment [7,8]. Together with their chemical complexity, the well validated pharmaceutical potential of sponge-derived natural products has sustained an interest in their discovery, structural description, chemical synthesis, and bioactivity characterization [9,10].

Sponges are holobionts, comprised of the eukaryotic sponge host with an associated prokaryotic microbiome. Simplistically, the sponge microbiome can be grouped into two parts, the symbiotic and the commensal microbiome. For high microbial abundance sponges, the symbiotic microbiome can account for up to 40% of the sponge biomass [11,12]. In numerous cases, the symbiotic bacteria have been demonstrated to be the physiological producers of the bioactive natural products that were isolated from sponge tissues [13,14]. Untargeted metagenomic studies have revealed additional genetic potential in the symbiotic microbiome for the production of as yet unknown cryptic natural products [15,16,17]. Due to the obligate nutritional and structural support that the symbiotic microbiome conceivably derives from the holobiont matrix, these sponge symbiotic bacteria frequently resist laboratory cultivation [18]. Hence, access to the natural products produced by the sponge symbiotic microbiome relies on the isolation of these natural products from sponge tissues, via heterologous expression of the biosynthetic genes and gene clusters, or via chemical synthesis.

In addition to the symbiotic microbiome, marine sponges are also associated with putatively commensal bacteria that are amenable to cultivation in the laboratory. The exact nature of the relationship between the non-obligate symbiotic microbial community and the sponge may be complex and variable. However, network analysis revealed that amensal or commensal interactions within the sponge are more prevalent than mutualistic or competitive interactions [19]; thus, herein, we will refer to this bacterial community as commensal. Isolation of bioactive natural products from these commensal bacteria establishes them as sources of chemical novelty and potentiates their pharmacological utility. However, unlike the symbionts wherein the metabolomic overlap between the sponge holobiont and the symbiotic bacteria is now abundantly well established, it has not yet been mapped out whether the commensal bacteria produce metabolites and natural products that are also detected in the sponge extracts themselves. By large scale culturing of commensal bacteria from three different high microbial abundance sponges, in this study, we establish that the metabolomic overlap between the commensal bacteria and the sponge holobiont is minimal. We also find that the phylogeny of the cultured commensal bacteria is disparate from that of the symbiotic microbiome and that the metabolomic architectures of these commensal bacteria are largely independent of the source sponges. By annotation of the mass spectrometry fragmentation data, we present evidence for the presence of novel natural products in the extracts of commensal bacteria and provide insights into their chemical identities with an aim to facilitate isolation and detailed structure elucidation studies.

The findings described herein are derived from an undergraduate laboratory course that was developed and implemented in the School of Biological Sciences at Georgia Institute of Technology (Georgia Tech) in Fall 2022. Pedagogic research has established that exposing undergraduate students to a higher-level experimental coursework is key in delivering enhanced intellectual outcomes [20,21,22,23]. However, even at relatively well-equipped institutions such as Georgia Tech, there are hardly any courses for undergraduate students that deliver hands-on experimental instruction in contemporary tools in metabolomics and natural product chemistry. Furthermore, the COVID-19 pandemic has had a profound deleterious impact on undergraduate research [24,25,26,27]. It is thus our motivation that the findings and workflows described herein will motivate the development of similar curricula at other institutions, ultimately seeding interest in undergraduate students for continuing to engage with natural products and -omics-based research at the postgraduate levels and beyond.

## 2. Results and Discussion

### 2.1. Marine Sponge Specimens

**Figure 1:**
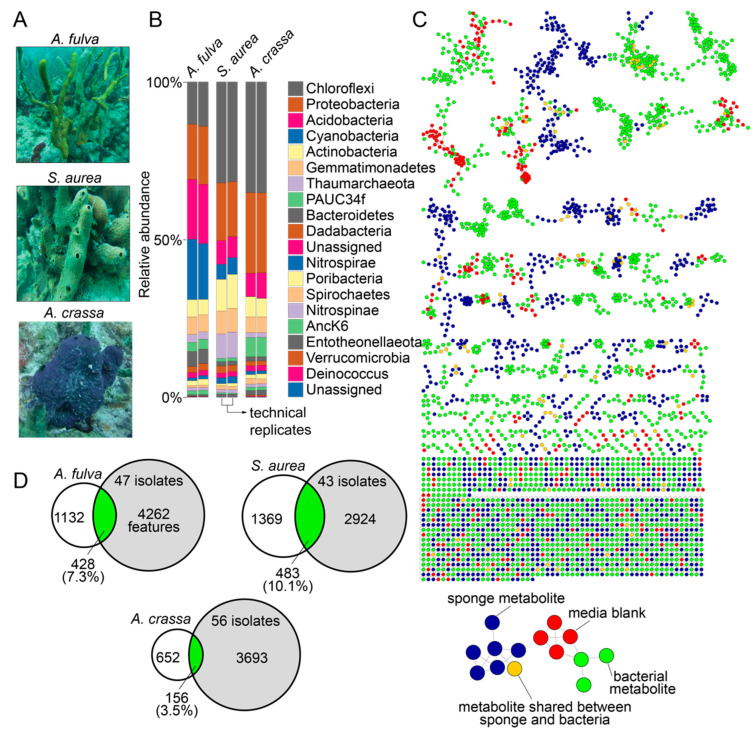
Microbiomes and metabolomes of sponges and bacterial isolates. (**A**) Morphology of the Floridian sponges *A. fulva*, *S. aurea*, and *A. crassa*. (**B**) Phylum-level microbiome architectures of the three sponge holobionts queried in technical duplicates (data reported in Ref. [16]). (**C**) Molecular network illustrating the structural similarity between metabolites detected in sponge tissue (blue nodes), bacterial isolates (green nodes), and metabolites that were shared between sponge tissue and bacterial isolates (brown nodes). Metabolites detected in media used for bacterial cultivation, and those between media and sponge tissue and media and bacterial isolates are represented as red nodes. (**D**) After subtraction of metabolites detected in media, the overlaps between metabolites detected in bacterial isolates (gray circles) and sponge tissues (white circles) are illustrated as Venn diagrams.

In this study, we used specimens of marine sponges *Aplysina fulva*, *Smenospongia aurea*, and *Aiolochroia crassa* that are abundantly present on the shallow reefs in Florida Keys (Figure 1A). We have described the phylogenetic identification of these sponge specimens using Sanger sequencing of amplicons of the 28S rRNA gene and the internal transcribed space 2 (ITS-2) previously, and our choice to use these sponges in this study was dictated by our prior characterization of their microbiome and metabolome architectures [16,28,29]. The diversity of the bacterial phyla present in the sponge holobionts and with the abundance of bacteria of the phylum Chloroflexi imply that these sponges are high microbial abundance sponges (Figure 1B) [30,31]. Furthermore, we have extensively mined the metabolomes of these sponges using liquid chromatography/mass spectrometry (LC/MS) with dereplication of numerous natural product chemical classes. Taken together, the abundance of these sponges in the Floridian reefs, the high microbial abundance in these sponge species, and our ability to rapidly dereplicate natural products from these sponges using mass spectrometry make these specimens well suited for this study.

### 2.2. Metabolomes of Sponges and Bacterial Isolates

Sponge tissues were minced and clarified homogenates were spread on three different solid media containing Petri dishes with and without the presence of antifungal nystatin and the antibiotic nalidixic acid to prevent the growth of fungi and fast-growing Gram-negative bacteria that would otherwise outgrow slower growing bacteria. Serial culturing of isolated colonies was performed on fresh Petri dishes and was sequentially repeated until morphologically pure cultures were obtained. Petri dishes possessing bacterial colonies with multiple different morphologies after three serial passages were discarded, as were any Petri dishes that demonstrated fungal contamination. Using three different growth media, 47, 43, and 56 bacterial isolates were obtained from the sponges *A. fulva*, *S. aurea*, and *A. crassa*, respectively, totaling 146 bacterial isolates derived from three marine sponges. The growth conditions for each bacterial isolate are described in Supplementary Table S1.

Bacterial culture extracts, sponge tissue extracts, and extracts of blank media used for bacterial cultivation were analyzed by LC/MS. Data thusly generated were organized in a network format using the online Global Natural Product Social Molecular Networking (GNPS) platform (Figure 1C) [32]. Numerous sponge-derived natural product chemical classes were dereplicated by comparison to spectral libraries. These include the bromotyrosine alkaloids (such as the purealidins and the aerophobins; detected in *A. fulva* and *A. crassa*) [29,33], the bromotryptophan alkaloids (such as bromotryptamines and tubastrindoles; detected in *S. aurea*) [28,34], the hybrid peptide/polyketide natural products smenamides and smenathiazoles (detected in *S. aurea*) [28,35,36], and the drimane meroterpenes (such as aureol and cyclosmenospongine; detected in *S. aurea*) [37,38]. In addition to dereplicated natural products, it was abundantly clear that the chemical diversity in marine sponge extracts detected in these mass spectrometry data was much greater than that had been realized by isolation and structure elucidation of individual natural products; an observation that we have previously realized for other marine sponges as well [14,39,40]. It is noteworthy that all above-mentioned natural products were detected to be present in sponge tissues only and were not detected to be present in extracts of bacterial isolates. Dereplication of natural products and metabolites detected in bacterial extracts was sparse and was restricted to phospholipids, fatty acids, amino acids, and siderophores. A majority of bacteria-derived clusters in the molecular network illustrated in Figure 1C not possessing any dereplicated nodes points towards the potential of bacterial extracts described in this study as sources of novel metabolites and natural products (vide infra).

Metabolomic features from the LC/MS data were extracted using MZmine [41]. After removal of features detected in media blanks, the metabolomic overlap between sponge tissue extracts and extracts of bacterial isolates ranged from 3.5% (between *A. crassa* and 56 bacterial strains isolated from *A. crassa*) to 10.1% (between *S. aurea* and 43 bacterial strains isolated from *S. aurea*, Figure 1D). A majority of these shared metabolites between bacteria and sponges, illustrated as brown nodes in Figure 1C, were phospholipids and fatty acids. That unculturable obligate bacterial symbionts synthesize a large fraction of the natural products detected in sponge holobionts is now well established [13,18,42]. Data presented in this study allow us to posit that laboratory culturing-amenable commensal bacteria have a much lower contribution to the overall metabolome of the marine sponge holobiont. Nonetheless, these commensal bacteria remain an important source for the discovery of novel and potentially bioactive natural products.

### 2.3. Phylogeny of Bacterial Isolates

Sanger sequencing of full length 16S rRNA gene amplicons was used to assign the genera for bacterial strains isolated in this study. Consensus genera assignment relied upon comparison of forward and reverse 16S rRNA gene amplicon Sanger sequences to the GenBank rRNA/ITS database using the Basic Local Alignment Search Tool (BLAST) (Supplementary Table S1) [43].

**Figure 2:**
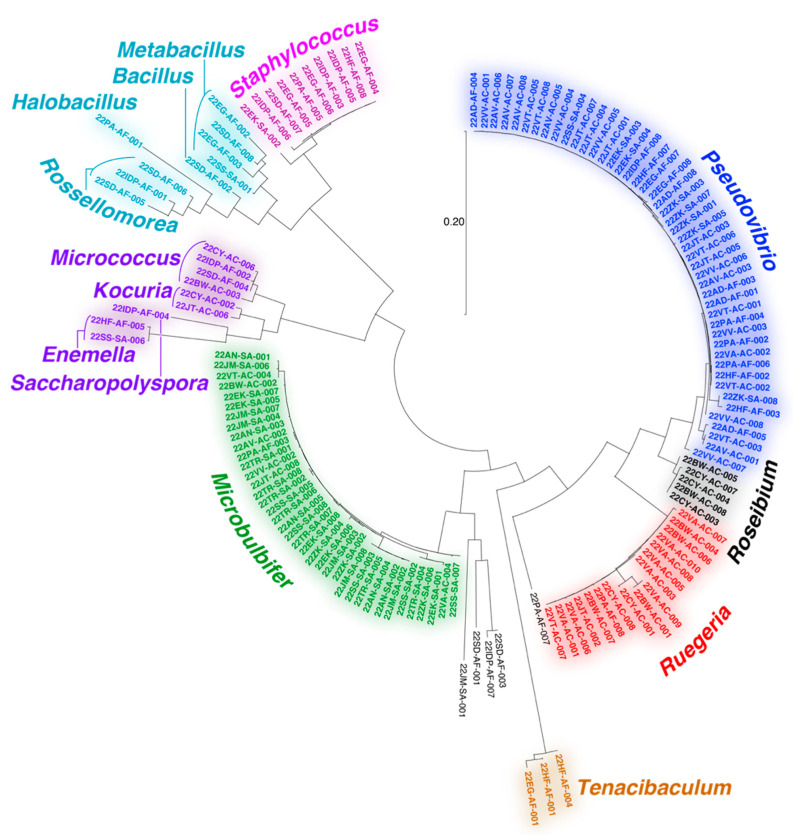
Dendogram representing 16S rRNA gene relatedness among bacteria isolated in this study. Bacteria genera are labeled.

The majority of bacteria isolated in this study could be assigned to the Gram-negative proteobacterial *Pseudovibrio*, *Ruegeria*, and *Microbulbifer* genera (Figure 2). All three genera have extensive literature precedents for association with marine invertebrates and are well validated sources of bioactive natural products [44,45,46,47,48,49,50,51]. Recently, a *Pseudovibrio* isolate from a Verongid sponge was reported to produce bromotyrosine alkaloid natural products [52]. However, bromotyrosine alkaloids were not detected in extracts of any of the *Pseudovibrio* strains isolated in this study. Other noteworthy bacterial isolates include actinomycetes belonging to the genera *Micrococcus*, *Kocuria*, *Enemella*, and *Saccharopolyspora* and bacilli of the genera *Bacillus*, *Metabacillus*, *Halobacillus*, and *Rossellomorea*. We also detected the isolation of strains from *Tenacibaculum*, a genus of opportunistic fish pathogenic bacteria. The diversity and genera of bacteria isolated in this study agree with prior studies describing cultivation of bacteria from marine sponges [53,54,55].

Overall, the diversity of culturing-amenable commensal bacteria was lesser than that of symbiotic bacteria associated with the sponge holobiont (Figure 1B and Figure 2). Numerous phyla abundantly present in the sponge microbiome, such as Chloroflexi and Acidobacteria, are not represented in the cultured commensal bacteria; this observation is in line with findings from other sponges such as the giant barrel *Xestospongia* spp. sponges [56]. While it can be rationalized that the culturing conditions used in this study were not amenable to isolation of cyanobacteria and members of sponge-specific phyla such as Poribacteria and Entotheonellaeota, data presented here allow us to establish that the commensal bacterial community associated with marine sponges is vastly different in phylogenetic identity and metabolomic potential as compared to the sponge symbiotic microbiome.

### 2.4. Dependence of Isolation Media and Source Sponge on Bacterial Metabolomes

**Figure 3:**
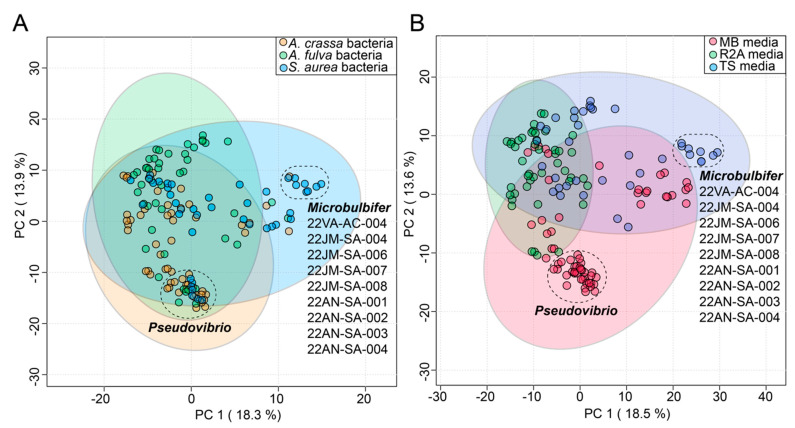
Effect of (**A**) sponge sources and (**B**) cultivation media on diversity of commensal bacterial metabolomes as visualized using PCA plots. The variance explained by each component is labeled on the respective axes. The 95% confidence ellipses are included on the plots. Two distinct clusters of bacterial nodes are labeled in both plots, comprising of *Pseudovibrio* strains isolated from different sponges and *Microbulbifer* strains isolated mainly from *S. aurea*. The *Microbulbifer* strains are listed for clarity.

In this study, the commensal bacteria were cultured from three sponges using three different media. To gauge the influence, if any, of the source sponge and of the isolation media on the metabolomes of commensal bacteria, the bacterial metabolomes were visualized using principal component analysis (PCA) plots. The circular nodes on these plots represent metabolomes of individual bacteria (Figure 3).

Identity of the source sponges had minimal impact on bacterial metabolomes; metabolomes of most of the commensal bacteria cluster together on the first principal component (PC) in Figure 3A. This observation is perhaps best exemplified by a tight cluster on the first and second PCs of *Pseudovibrio* nodes that contains strains derived from all three sponges. The only discernable separated cluster on the first PC was that of nine *Microbulbifer* strains. Curiously, eight of these nine *Microbulbifer* strains were isolated from *S. aurea*– the mechanistic reason for this divergence is not immediately clear.

The cultivation media had a relatively larger impact on the separation of the bacterial metabolomes (Figure 3B). The use of Marine Broth 2216 (abbreviated as ‘MB media’ in Figure 3B) was strongly correlated with the isolation of *Pseudovibrio* strains from all three sponges (Supplementary Table S1); this observation is also reflected in the PCA plots where the impact of the MB media on the metabolomes of these strains is captured in the second PC (Figure 3B). The abovementioned nine *Microbulbifer* strains were all isolated using the tryptic soy media (abbreviated as ‘TS media’ in Figure 3B). Taken together, these results allow us to posit that innovation in culturing techniques and media compositions will have a greater impact on increasing the diversity of cultured commensal bacteria, as compared to attempts at increasing the marine invertebrate metazoan sample space itself.

### 2.5. Cryptic Metabolites Are Shared between Sponges and Commensal Bacteria

**Figure 4:**
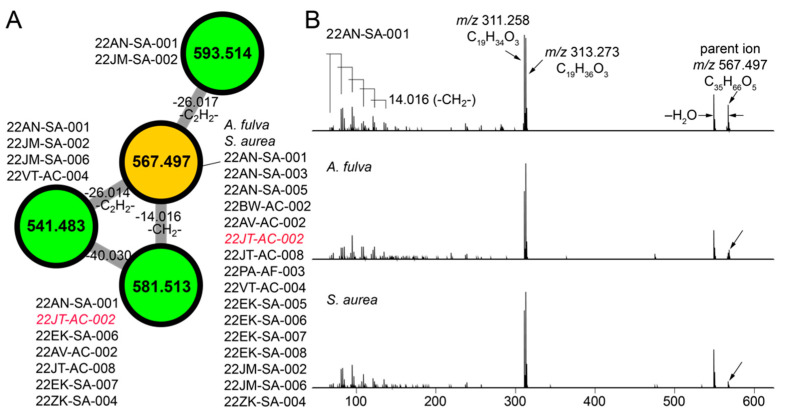
Discovery of cryptic metabolites shared between marine sponges and commensal bacteria. (**A**) A cluster of four nodes in which three nodes (in green) are detected in commensal bacteria only, and one node (in brown) is shared between commensal bacteria and *A. fulva* and *S. aurea* sponges. The parent masses for metabolites corresponding to the four nodes are labeled. For the *m/z* 567.497 node, (universal spectrum identifier (USI) 42 spectra were collated from commensal bacteria, and 16 spectra were collated from marine sponges. USI links for other nodes are as follows: *m/z* 541.483 node USI; *m/z* 581.513 node USI; *m/z* 593.514 node. (**B**) MS^2^ spectra for the *m/z* 567.497 metabolite, as detected in highest abundance in the *Microbulbifer* strain 22AN-SA-001 (top), and the *A. fulva* and *S. aurea* sponges (middle and bottom, respectively). The molecular formulae of the parent ions, and the two most abundant daughter ions, *m/z* 311.258 and *m/z* 313.273 are labeled. At low *m/z*, a number of daughter ions separated by 14.016 Da are discernable which corresponds to differences in a methylene (-CH_2_-) unit.

The molecular network illustrated in Figure 1C allowed us to mine for metabolites that are shared between sponge and commensal bacteria. Here, we disregarded phospholipids as they presumably represent cell wall components rather than secondary metabolites. For the cluster illustrated in Figure 4A, we observed three nodes (in green) derived from commensal bacteria connected to a node representing a metabolite with the parent mass *m/z* 567.497 that was shared between *A. fulva* and *S. aurea* sponges and sixteen commensal bacteria (in brown, Figure 4A). Of these sixteen commensal bacterial strains, all but one of the strains (22JT-AC-002, in red in Figure 4A) belong to the *Microbulbifer* genus. Nodes connected to this central node which represent metabolites detected only in bacterial extracts were similarly derived from *Microbulbifer* strains. Mass differences between the four nodes can be interpreted in chemically meaningfully terms, as labeled on the edges in Figure 4A.

Next, the MS^2^ spectra for the *m/z* 567.497 metabolite detected in the sponge extracts and the bacterial extracts were compared to reveal highly similar fragmentation patterns (Figure 4B). From the MS^2^ spectra, two highly abundant fragments with *m/z* 311.258 and *m/z* 313.273 were identified to which the molecular formulae C_19_H_34_O_3_ and C_19_H_36_O_3_ were assigned, respectively. In addition, a series of smaller fragment ions differing by 14.016 Da were observed which were suggestive of a long, saturated hydrocarbon appendage. The molecular formula of the *m/z* 567.497 parent ion, C_35_H_66_O_5_, denoted three degrees of unsaturation. With these structural insights, attempts at dereplication using the MarinLit and NPAtlas [57] databases were unsuccessful, thus representing a novel metabolite/natural product shared between marine sponges and commensal *Microbulbifer* bacteria. Here, organization of the metabolomic data in the form of molecular networks allows for prioritization of candidate molecules for future isolation efforts, and the curation of fragmentation spectra provides valuable structural insights.

### 2.6. Commensal Bacteria Are Sources of Novel Natural Products

The numerous clusters in Figure 1C that contain nodes corresponding to metabolites produced only by commensal bacteria and which could not be dereplicated by GNPS, MarinLit, or NPAtlas likely represent novel metabolites and natural products. It is thus abundantly clear that the library of commensal bacteria generated as a part of this study can serve as a resource for generating chemical novelty. One such example is presented below.

**Figure 5:**
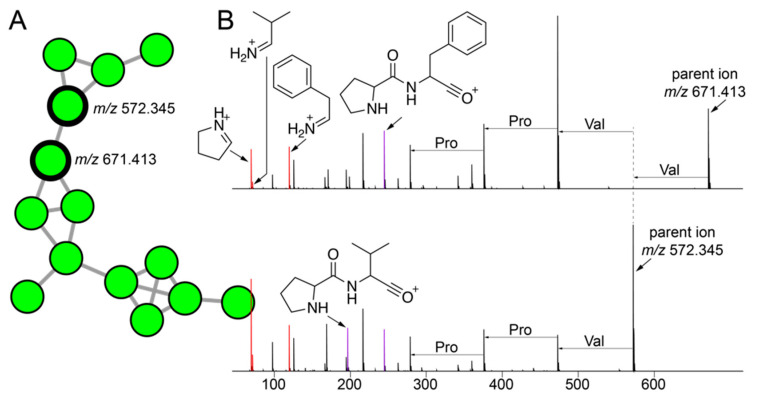
Cryptic peptidic natural products mined from commensal bacteria. (**A**) A cluster of nodes corresponding to metabolites detected only in extracts of commensal bacteria is shown with two nodes with the maximum number of MS^2^ spectra associated with them in this cluster labeled with their respective parent ion masses. (**B**) MS^2^ fragmentation spectra for metabolites with parent masses *m/z* 671.413 (top; USI and *m/z* 572.345 (bottom; USI). The proline, valine, and phenylalanine immonium ions (*m/z* 70.065, *m/z* 72.028, and *m/z* 120.081, respectively) are highlighted in red. The Pro-Val and Pro-Phe dipeptide oxonium ions (*m/z* 197.128 and *m/z* 245.128, respectively) are highlighted in purple. Progressing from the parent ions, mass differences between MS^2^ fragment ions corresponding to proteinogenic amino acids are labeled.

The cluster of nodes illustrated in Figure 5A were derived from a number of strains isolated using tryptic soy media from *A. fulva* and *A. crassa* sponges. As mentioned above, the bacterial isolation media had a larger differentiating effect on bacterial metabolomes than the sponge source. Among these nodes, two nodes with parent masses *m/z* 671.413 and *m/z* 572.345 had a much greater number of MS^2^ spectra (80 and 71 spectra, respectively) associated with them as compared to other nodes in the cluster (less than 5). Hence, we directed our efforts towards gleaning structural insights for metabolites corresponding to these two nodes. The highest abundance of both metabolites was detected in the extracts of the *Metabacillus* sp. bacterium 22SD-AF-008 (Figure 2 and Supplementary Table S1).

The MS^2^ spectra for both metabolites demonstrated immonium ions corresponding to the proteinogenic amino acids proline, valine, and phenylalanine (ions highlighted in red, Figure 5B). This observation was highly suggestive of the peptidic nature of the two metabolites. Furthermore, we annotated the Pro-Phe and Pro-Val dipeptide oxonium ions (highlighted in purple, Figure 5B). As we have recently demonstrated for proline containing peptidic natural products from marine sponges [58], the amide bonds at the N-termini of proline residues are highly susceptible to cleavage in a mass spectrometry-based fragmentation experiment which facilitates the formation of the Pro-Xaa dipeptide oxonium fragment ions [59,60]. Furthermore, we annotated mass differences corresponding to proteinogenic amino acids between MS^2^ fragment ions in both spectra (Figure 5B). The amino acid identities as discerned by these mass differences, proline, and valine, are supported by the detection of the abovementioned immonium ions. It should be noted that unlike for proline-rich macrocyclic peptides detected in marine sponges, the entire MS^2^ spectra could not to be annotated as a continuous string of mass differences corresponding only to proteinogenic amino acids. Hence, as yet unknown modifications to either amino acid side chains or to the polyamide backbones are present in these two natural products. As before, these structural insights will facilitate a thorough structural characterization using spectroscopic and crystallographic experiments in the future. Future efforts will also be directed towards connecting these peptidic natural products to their corresponding biosynthetic gene clusters.

### 2.7. Pedagogic Deliverables

During the course of these studies, eighteen undergraduate students in the School of Biological Sciences at Georgia Tech received hands-on instruction in bacterial culturing, derivation of axenic strains from complex matrices, liquid–liquid extraction of bacterial cultures, genomic DNA isolation from bacterial cultures, and mass spectrometry data annotation and visualization using GNPS and MZmine. These are valuable skill sets in the contemporary environment of natural products research wherein -omics-based tools and technologies are gaining increasing prominence. With three source sponges and three isolation media, the eighteen student participants were divided in teams of two, with each team focused on derivation of axenic strains from a particular sponge using a single isolation media. All experiments were conducted with attention to chemical and biological safety.

The data and findings developed as a part of this effort are described with the motivation that similar efforts will enrich the overall pool of postgraduate students with interest in natural products research and with prior training in bacterial culturing and mass spectrometry-based metabolomics, together with a theoretical grounding for the need for such efforts in the era where the efficacy of clinically used antibiotics and pharmaceuticals is decreasing. It should be noted that the democratization and free access to tools and databases such as GNPS, MZmine, and NPAtlas, in addition to access to advanced instrumentation is critical to the realization of such efforts, as has been demonstrated for similar pedagogic efforts reported in literature [61,62,63,64,65,66].

## 3. Materials and Methods

### 3.1. Collection of Sponge Specimens

Replicate individuals of sponges *A. fulva*, *S. aurea*, and *A. crassa* were collected from a shallow (5–7 m) coral reef off the coast of Summerland Key in the Florida Keys (coordinates 24.562811 and −81.403737). Portions of each species were removed from a parent sponge using a razor blade and held in individual bags underwater. On the boat, sponges were held on ice for transport back to the laboratory for processing. Sponges were collected under Florida Keys National Marine Sanctuary (FKNMS) permit FKNMS-2021-049 to C.G.E.

### 3.2. Bacterial Culturing

Sponge specimens were washed extensively with Tris-EDTA buffer (TE buffer; 10 mM Tris-Cl (pH 8.0), 1 mM ethylenediaminetetraacetic acid sodium salt (EDTA-Na)) and minced with sterile razor blades. The slurry thusly obtained was diluted 10-fold with TE buffer and centrifuged at 500× *g* for 2 min in a microcentrifuge to remove debris. The supernatant was diluted 2-fold with sterile 40% *v*/*v* glycerol and stored at −80 °C in small aliquots.

Aliquots of the thusly prepared sponge exudates were thawed on ice. Serial dilutions (1×; 1/10×; and 1/100×) were plated on MB-agar, TS-agar, and R2A-agar media prepared in artificial sea water (ASW) with the following compositions:

ASW: 26.29 g/L NaCl, 0.74 g/L KCl, 0.99 g/L CaCl_2_, 6.09 g/L MgCl_2_.6H_2_O, 3.94 g/L MgSO_4_.7H_2_O, 1.0 g/L KBr, and 0.2 g/L KI; filtered and autoclaved

MB-agar: 18.7 g/L marine broth 2216, 15 g/L agar, 50% *v*/*v* ASW; autoclaved

TS-agar: 30.0 g/L tryptic soy broth, 15 g/L agar, 50% *v*/*v* ASW; autoclaved

R2A-agar: 3.2 g/L R2A broth, 15 g/L agar, 50% *v*/*v* ASW; autoclaved

Individual colonies from petri dishes plated with sponge exudates were re-streaked onto fresh petri dishes. This procedure was serially repeated thrice, until morphologically uniform colonies without fungal contamination were obtained. For metabolomics and DNA isolation, single bacterial colonies were inoculated in liquid media with identical composition as above but with agar omitted.

### 3.3. Preparation of Extracts for LC/MS

Liquid media were inoculated with bacteria and allowed to grow for 5–7 days with shaking (media and growth temperatures listed in Supplementary Table S1). Then, 5 mL liquid cultures were extracted twice with equal volume of ethyl acetate. The organic layers were pooled, and the organic solvent was removed in vacuo. The extract was then resuspended in 1 mL methanol with sonication and clarified by centrifugation. The clarified extracts were then used for LC/MS data collection. Extracts of cultivation media were generated in an identical fashion.

Sponge tissues were frozen and lyophilized to dryness. The dried sponge tissues were crushed and extracted in 1:1 dichloromethane/methanol (1 mL solvent/5 mg biomass) for 48 h with agitation. The solvent was filtered and dried in vacuo. The extracts were resuspended in methanol with sonication and clarified by centrifugation. The clarified extracts were used for LC/MS data collection.

### 3.4. LC/MS Data Collection, Preprocessing, and Statistical Analysis

All samples were analyzed with an Agilent 1290 Infinity II UHPLC system (Agilent Technologies, Santa Clara, CA, USA) using a Kinetex 1.7 μm C18 reverse-phase UHPLC column (50 × 2.1 mm, Phenomenex, Torrance, CA, USA) coupled to an ImpactII ultrahigh resolution Qq-ToF mass spectrometer (Bruker Daltonics, Billerica, MA, USA) equipped with an electrospray ionization (ESI) source for a mass spectrometry (MS/MS) analysis [67]. MS spectra were acquired in the positive mode with an *m*/*z* range of 50–2000 Da. The eight most intense ions per MS^1^ spectra were selected for further acquisition of MS^2^ data. An active exclusion of two spectra was used, implying that an MS^1^ ion would not be selected for fragmentation after two consecutive MS^2^ spectra had been recorded for it in a 0.5 min time window. The exclusion was reconsidered, and an additional MS^2^ spectrum was acquired if a fivefold enhancement in intensity was observed. The chromatography solvent A: H_2_O (Fisher Chemical, LC/MS grade) + 0.1% *v*/*v* formic acid (Fisher Scientific, Waltham, MA, USA LC/MS grade) and solvent B: MeCN (Fisher Chemical, LC/MS grade) + 0.1% *v*/*v* formic acid were employed for separation. The flow rate was held constant at 0.5 mL/min throughout. The gradient applied for chromatographic separation was 5% solvent B and 95% solvent A for 3 min, a linear gradient of 5% B–95% B over 17 min, held at 95% B for 3 min, 95% B–5% B in 1 min, and held at 5% B for 1 min, 5% B–95% B in 1 min, held at 95% B for 2 min, 95% B–5% B in 1 min, then held at 5% B for 2.5 min. Following the acquisition of data on 12 samples, data on a mixture of six compounds (amitryptiline, sulfamethazine, sulfamethizole, sulfachloropyridazine, sulfadimethoxine, coumarin-314) were acquired as a quality control step to ensure consistent instrument and column performance.

The raw data were converted to the mzXML format using vendor software. Metabolite features were extracted using MZmine to perform steps for mass detection, chromatogram building, chromatogram deconvolution, isotopic grouping, retention time alignment, duplicate removal, and missing peak filling [41]. Prior to statistical analysis, blank subtraction was performed as previously described [68] on the quantification tables using a Jupyter notebook, available at GitHub–https://github.com/Garg-Lab/Sponge-and-dervied-Bacteria-Blank-Subtraction-2022 (accessed on 11 January 2023). The filtered quantification table were submitted to MetaboAnalyst 5.0 and log transformation was used for normalization prior to principle component analysis [69]. Cytoscape was used to visualize the molecular network generated on GNPS and to generate Venn diagrams [70].

### 3.5. Genomic DNA Isolation

Genomic DNA from bacterial cultures was isolated using the GeneJET Genomic DNA Purification Kit (Thermo Fisher Scientific, Waltham, MA, USA) and manufacturer’s protocols with the assumption that all bacterial strains were Gram positive. Hence, a bacterial cell lysis buffer comprising of 20 mM Tris-Cl (pH 8.0), 2 mM EDTA-Na, 1.2% (*v*/*v*) of Triton X-100, and 20 mg/mL lysozyme was used. No modifications to the manufacturer’s protocol were made.

### S rRNA Gene Amplicon Sequencing

PCR amplification of the full length 16S rRNA gene was performed in a 25 μL total reaction volume containing the following components: 12.5 ng of bacterial genomic DNA, Platinum II Hot-Start PCR Master Mix (2×) (Thermo Fisher Scientific), and 0.2 μM of the following primers set: 27F- 5′ AGRGTTYGATYMTGGCTCAG -3′ and 1492R- 5′- RGYTACCTTGTTACGACTT- 3′. PCR reactions were performed on the Applied Biosystem ProFlex PCR system using the following program: 95 °C for 3 min, followed by 30 cycles of 95 °C for 30 s, 55 °C for 30 s, and 72 °C for 1 min, and a final extension at 72 °C for 5 min. PCR products were then purified using the Agencourt AMPure XP beads (Beckman Coulter, Brea, CA, USA) to remove contaminants, unused primers, and primer dimers, among other impurities. The size of the PCR amplicons was verified on the Agilent Bioanalyzer-High Sensitivity chip before Sanger sequencing.

Sanger sequencing reactions were performed using the BigDye Terminator v3.1 Cycle Sequencing Kit (Applied Biosystems, Waltham, MA, USA) following a slightly modified manufacturer protocol. Two reactions were run per sample: a forward and the reverse. The BigDye reaction was carried out in a 10 μL final volume by combining 1 μL purified PCR products (10–40 ng), 1.55 µL of the 5 × BigDye Terminator v3.1 Sequencing Buffer, 1 µL of 3.2 µM Forward or reverse primer, 0.5 µL of the BigDye Terminator, 5.95 µL of water. The samples were run on the Applied Biosystem ProFlex Thermocycler using the following program: 96 °C for 1 min, followed by 25°ycles of 96 °C for 10 s, 50 °C for 5 s, and extension at 60 °C for 4 min. DNA sequencing reactions were purified using magnetic beads to remove unincorporated BigDye terminators and salts and then sequenced on the Applied Biosystems SeqStudio Flex.

## Data Availability

The LC-MS/MS datasets generated in this study are available in the public repository Mass spectrometry Interactive Virtual Environment (MassIVE) at https://massive.ucsd.edu/ (accessed on 11 January 2023) with the identifier MSV000090609.

## Supplementary Materials

The following supporting information can be downloaded at: https://www.mdpi.com/article/10.3390/md21010053/s1, Table S1: bacterial strain repository.

## References

[1] de Goeij J.M., van Oevelen D., Vermeij M.J.A., Osinga R., Middelburg J.J., de Goeij A.F.P.M., Admiraal W. (2013). Surviving in a marine desert: The sponge loop retains resources within coral reefs. Science.

[2] McMurray S.E., Stubler A.D., Erwin P.M., Finelli C.M., Pawlik J.R. (2018). A test of the sponge-loop hypothesis for emergent Caribbean reef sponges. Mar. Ecol. Prog. Ser..

[3] Bart M.C., Hudspith M., Rapp H.T., Verdonschot P.F.M., de Goeij J.M. (2021). A deep-sea sponge loop? Sponges transfer dissolved and particulate organic carbon and nitrogen to associated fauna. Front. Mar. Sci..

[4] Loh T.-L., Pawlik J.R. (2014). Chemical defenses and resource trade-offs structure sponge communities on Caribbean coral reefs. Proc. Natl. Acad. Sci. USA.

[5] Helber S.B., Hoeijmakers D.J.J., Muhando C.A., Rohde S., Schupp P.J. (2018). Sponge chemical defenses are a possible mechanism for increasing sponge abundance on reefs in Zanzibar. PLoS ONE.

[6] Puglisi M.P., Sneed J.M., Ritson-Williams R., Young R. (2019). Marine chemical ecology in benthic environments. Nat. Prod. Rep..

[7] Abdelaleem E.R., Samy M.N., Desoukey S.Y., Liu M., Quinn R.J., Abdelmohsen U.R. (2020). Marine natural products from sponges (Porifera) of the order Dictyoceratida (2013 to 2019); a promising source for drug discovery. RSC Adv..

[8] Carroll A.R., Copp B.R., Davis R.A., Keyzers R.A., Prinsep M.R. (2021). Marine natural products. Nat. Prod. Rep..

[9] Gerwick W.H., Moore B.S. (2012). Lessons from the past and charting the future of marine natural products drug discovery and chemical biology. Chem. Biol..

[10] Jiménez C. (2018). Marine natural products in medicinal chemistry. ACS Med. Chem. Lett..

[11] Hentschel U., Piel J., Degnan S.M., Taylor M.W. (2012). Genomic insights into the marine sponge microbiome. Nat. Rev. Microbiol..

[12] Gloeckner V., Wehrl M., Moitinho-Silva L., Gernert C., Schupp P., Pawlik J.R., Lindquist N.L., Erpenbeck D., Wörheide G., Hentschel U. (2014). The HMA-LMA dichotomy revisited: An electron microscopical survey of 56 sponge species. Biol. Bull..

[13] Morita M., Schmidt E.W. (2018). Parallel lives of symbionts and hosts: Chemical mutualism in marine animals. Nat. Prod. Rep..

[14] Paul V.J., Freeman C.J., Agarwal V. (2019). Chemical ecology of marine sponges: New opportunities through “-omics”. Integr. Comp. Biol..

[15] Lackner G., Peters E.E., Helfrich E.J., Piel J. (2017). Insights into the lifestyle of uncultured bacterial natural product factories associated with marine sponges. Proc. Natl. Acad. Sci. USA.

[16] Nguyen N.A., Lin Z., Mohanty I., Garg N., Schmidt E.W., Agarwal V. (2021). An obligate peptidyl brominase underlies the discovery of highly distributed biosynthetic gene clusters in marine sponge microbiomes. J. Am. Chem. Soc..

[17] Cahn J.K.B., Piel J. (2021). Opening up the single-cell toolbox for microbial natural products research. Angew. Chem. Int. Ed..

[18] Simmons T.L., Coates R.C., Clark B.R., Engene N., Gonzalez D., Esquenazi E., Dorrestein P.C., Gerwick W.H. (2008). Biosynthetic origin of natural products isolated from marine microorganism-invertebrate assemblages. Proc. Natl. Acad. Sci. USA.

[19] Thomas T., Moitinho-Silva L., Lurgi M., Björk J.R., Easson C., Astudillo-García C., Olson J.B., Erwin P.M., López-Legentil S., Luter H. (2016). Diversity, structure and convergent evolution of the global sponge microbiome. Nat. Commun..

[20] Bruck L.B., Towns M., Bretz S.L. (2010). Faculty Perspectives of Undergraduate Chemistry Laboratory: Goals and Obstacles to Success. J. Chem. Educ..

[21] Zwickl B.M., Finkelstein N., Lewandowski H.J. (2012). The process of transforming an advanced lab course: Goals, curriculum, and assessments. Am. J. Phys..

[22] Kerr M.A., Yan F. (2016). Incorporating Course-Based Undergraduate Research Experiences into Analytical Chemistry Laboratory Curricula. J. Chem. Educ..

[23] Velasco J.B., Knedeisen A., Xue D., Vickrey T.L., Abebe M., Stains M. (2016). Characterizing Instructional Practices in the Laboratory: The Laboratory Observation Protocol for Undergraduate STEM. J. Chem. Educ..

[24] Qiang Z., Obando A.G., Chen Y., Ye C. (2020). Revisiting distance learning resources for undergraduate research and lab activities during COVID-19 pandemic. J. Chem. Educ..

[25] Elmer S.J., Durocher J.J. (2020). Moving student research forward during the COVID-19 pandemic. Adv. Physiol. Educ..

[26] Sunasee R. (2020). Challenges of teaching organic chemistry during COVID-19 pandemic at a primarily undergraduate institution. J. Chem. Educ..

[27] Grineski S.E., Morales D.X., Collins T.W., Nadybal S., Trego S. (2021). Anxiety and depression among US college students engaging in undergraduate research during the COVID-19 pandemic. J. Am. Coll. Health.

[28] Cantrell T.P., Freeman C.J., Paul V.J., Agarwal V., Garg N. (2019). Mass spectrometry-based integration and expansion of the chemical diversity harbored within a marine sponge. J. Am. Soc. Mass Spectrom..

[29] Mohanty I., Tapadar S., Moore S.G., Biggs J.S., Freeman C.J., Gaul D.A., Garg N., Agarwal V. (2021). Presence of bromotyrosine alkaloids in marine sponges is independent of metabolomic and microbiome architectures. mSystems.

[30] Moitinho-Silva L., Steinert G., Nielsen S., Hardoim C.C.P., Wu Y.-C., McCormack G.P., López-Legentil S., Marchant R., Webster N., Thomas T. (2017). Predicting the HMA-LMA status in marine sponges by machine learning. Front. Microbiol..

[31] Bayer K., Jahn M.T., Slaby B.M., Moitinho-Silva L., Hentschel U. (2018). Marine sponges as *Chloroflexi* hot spots: Genomic insights and high-resolution visualization of an abundant and diverse symbiotic clade. mSystems.

[32] Wang M.X., Carver J.J., Phelan V.V., Sanchez L.M., Garg N., Peng Y., Nguyen D.D., Watrous J., Kapono C.A., Luzzatto-Knaan T. (2016). Sharing and community curation of mass spectrometry data with Global Natural Products Social Molecular Networking. Nat. Biotechnol..

[33] Peng J., Li J., Hamann M.T. (2005). The marine bromotyrosine derivatives. Alkaloids Chem. Biol..

[34] Bialonska D., Zjawiony K.J. (2009). Aplysinopsins—marine indole alkaloids: Chemistry, bioactivity and ecological significance. Mar. Drugs.

[35] Teta R., Irollo E., Della Sala G., Pirozzi G., Mangoni A., Costantino V. (2013). Smenamides A and B, chlorinated peptide/polyketide hybrids containing a dolapyrrolidinone unit from the Caribbean sponge *Smenospongia aurea*. Evaluation of their role as leads in antitumor drug research. Mar. Drugs.

[36] Via C.W., Glukhov E., Costa S., Zimba P.V., Moeller P.D.R., Gerwick W.H., Bertin M.J. (2018). The metabolome of a cyanobacterial bloom visualized by MS/MS-based molecular networking reveals new neurotoxic smenamide analogs (C, D, and E). Front. Chem..

[37] Djura P., Stierle D.B., Sullivan B., Faulkner D.J., Arnold E.V., Clardy J. (1980). Some metabolites of the marine sponges Smenospongia aurea and Smenospongia (.ident.Polyfibrospongia) echina. J. Org. Chem..

[38] Hu J.F., Schetz J.A., Kelly M., Peng J.N., Ang K.K.H., Flotow H., Leong C.Y., Ng S.B., Buss A.D., Wilkins S.P. (2002). New antiinfective and human 5-HT2 receptor binding natural and semisynthetic compounds from the Jamaican sponge *Smenospongia aurea*. J. Nat. Prod..

[39] Mohanty I., Moore S.G., Yi D., Biggs J.S., Gaul D.A., Garg N., Agarwal V. (2020). Precursor-guided mining of marine sponge metabolomes lends insight into biosynthesis of pyrrole–imidazole alkaloids. ACS Chem. Biol..

[40] Mohanty I., Podell S., Biggs S.J., Garg N., Allen E.E., Agarwal V. (2020). Multi-omic profiling of *Melophlus* sponges reveals diverse metabolomic and microbiome architectures that are non-overlapping with ecological neighbors. Mar. Drugs.

[41] Pluskal T., Castillo S., Villar-Briones A., Orešič M. (2010). MZmine 2: Modular framework for processing, visualizing, and analyzing mass spectrometry-based molecular profile data. BMC Bioinform..

[42] Bewley C.A., Faulkner D.J. (1998). Lithistid sponges: Star performers or hosts to the stars. Angew. Chem. Int. Ed..

[43] Boratyn G.M., Camacho C., Cooper P.S., Coulouris G., Fong A., Ma N., Madden T.L., Matten W.T., McGinnis S.D., Merezhuk Y. (2013). BLAST: A more efficient report with usability improvements. Nucleic Acids Res..

[44] Mitova M., Popov S., De Rosa S. (2004). Cyclic peptides from a *Ruegeria* strain of bacteria associated with the sponge *Suberites domuncula*. J. Nat. Prod..

[45] Quévrain E., Domart-Coulon I., Pernice M., Bourguet-Kondracki M.-L. (2009). Novel natural parabens produced by a *Microbulbifer* bacterium in its calcareous sponge host *Leuconia nivea*. Environ. Microbiol..

[46] Machado H., Sonnenschein E.C., Melchiorsen J., Gram L. (2015). Genome mining reveals unlocked bioactive potential of marine Gram-negative bacteria. BMC Genom..

[47] Naughton L.M., Romano S., O’Gara F., Dobson A.D.W. (2017). Identification of secondary metabolite gene clusters in the *Pseudovibrio* genus reveals encouraging biosynthetic potential toward the production of novel bioactive compounds. Front. Microbiol..

[48] Romano S. (2018). Ecology and biotechnological potential of bacteria belonging to the genus *Pseudovibrio*. Appl. Environ. Microbiol..

[49] Jayanetti D.R., Braun D.R., Barns K.J., Rajski S.R., Bugni T.S. (2019). Bulbiferates A and B: Antibacterial acetamidohydroxybenzoates from a marine proteobacterium, *Microbulbifer* sp. J. Nat. Prod..

[50] Karim M.R.U., Harunari E., Oku N., Akasaka K., Igarashi Y. (2020). Bulbimidazoles A–C, antimicrobial and cytotoxic alkanoyl imidazoles from a marine gammaproteobacterium *Microbulbifer* species. J. Nat. Prod..

[51] Ióca L.P., Dai Y., Kunakom S., Diaz-Espinosa J., Krunic A., Crnkovic C.M., Orjala J., Sanchez L.M., Ferreira A.G., Berlinck R.G.S. (2021). A family of nonribosomal peptides modulate collective behavior in Pseudovibrio bacteria isolated from marine sponges. Angew. Chem. Int. Ed..

[52] Nicacio K.J., Ióca L.P., Fróes A.M., Leomil L., Appolinario L.R., Thompson C.C., Thompson F.L., Ferreira A.G., Williams D.E., Andersen R.J. (2017). Cultures of the marine bacterium *Pseudovibrio denitrificans* Ab134 produce bromotyrosine-derived alkaloids previously only isolated from marine sponges. J. Nat. Prod..

[53] Sipkema D., Schippers K., Maalcke Wouter J., Yang Y., Salim S., Blanch Harvey W. (2011). Multiple approaches to enhance the cultivability of bacteria associated with the marine sponge *Haliclona* (*gellius*) sp. Appl. Environ. Microbiol..

[54] Esteves A.I.S., Amer N., Nguyen M., Thomas T. (2016). Sample processing impacts the viability and cultivability of the sponge microbiome. Front. Microbiol..

[55] Dat T.T.H., Steinert G., Cuc N.T.K., Smidt H., Sipkema D. (2021). Bacteria cultivated from sponges and bacteria not yet cultivated from sponges—A review. Front. Microbiol..

[56] Montalvo N.F., Davis J., Vicente J., Pittiglio R., Ravel J., Hill R.T. (2014). Integration of culture-based and molecular analysis of a complex sponge-associated bacterial community. PLoS ONE.

[57] van Santen J.A., Jacob G., Singh A.L., Aniebok V., Balunas M.J., Bunsko D., Neto F.C., Castaño-Espriu L., Chang C., Clark T.N. (2019). The Natural Products Atlas: An open access knowledge base for microbial natural products discovery. ACS Cent. Sci..

[58] Mohanty I., Nguyen N.A., Moore S.G., Biggs J.S., Gaul D.A., Garg N., Agarwal V. (2021). Enzymatic synthesis assisted discovery of proline-rich macrocyclic peptides in marine sponges. Chembiochem.

[59] Breci L.A., Tabb D.L., Yates J.R., Wysocki V.H. (2003). Cleavage N-terminal to proline:  analysis of a database of peptide tandem mass spectra. Anal. Chem..

[60] Kapp E.A., Schütz F., Reid G.E., Eddes J.S., Moritz R.L., O’Hair R.A.J., Speed T.P., Simpson R.J. (2003). Mining a tandem mass spectrometry database to determine the trends and global factors influencing peptide fragmentation. Anal. Chem..

[61] Petras D., Nothias L.F., Quinn R.A., Alexandrov T., Bandeira N., Bouslimani A., Castro-Falcon G., Chen L., Dang T., Floros D.J. (2016). Mass spectrometry-based visualization of molecules associated with human habitats. Anal. Chem..

[62] Betts T.A., Palkendo J.A. (2018). Teaching undergraduates LC–MS/MS theory and operation via multiple reaction monitoring (MRM) method development. J. Chem. Educ..

[63] Boyce M.C., Lawler N.G., Tu Y., Reinke S.N. (2019). Introducing undergraduate students to metabolomics using liquid chromatography–high resolution mass spectrometry analysis of horse blood. J. Chem. Educ..

[64] Kirk R.D., Carro M.A., Wu C., Aldine M.J., Wharton A.M., Goldstein D.G., Rosario M.E., Gallucci G.M., Zhao Y., Leibovitz E. (2021). Integrating natural product chemistry workflows into medicinal chemistry laboratory training: Building the PRISM library and cultivating independent research. J. Chem. Educ..

[65] Alker Amanda T., Gode Bhumika S., Aspiras Alpher E., Jones Jeffrey E., Michael Sama R., Aguilar D., Cain Audrea D., Candib Alec M., Cizmic Julian M., Clark Elise A. (2021). Draft genome sequences of 10 bacteria from the marine *Pseudoalteromonas* group. Microbiol. Resour. Announc..

[66] Winder C.L., Witting M., Tugizimana F., Dunn W.B., Reinke S.N., the Metabolomics Society Education and Training Committee (2022). Providing metabolomics education and training: Pedagogy and considerations. Metabolomics.

[67] Deutsch J.M., Mandelare-Ruiz P., Yang Y., Foster G., Routhu A., Houk J., De La Flor Y.T., Ushijima B., Meyer J.L., Paul V.J. (2022). Metabolomics Approaches to Dereplicate Natural Products from Coral-Derived Bioactive Bacteria. J. Nat. Prod..

[68] Deutsch J.M., Jaiyesimi O.A., Pitts K.A., Houk J., Ushijima B., Walker B.K., Paul V.J., Garg N. (2021). Metabolomics of Healthy and Stony Coral Tissue Loss Disease Affected *Montastraea cavernosa* Corals. Front. Mar. Sci..

[69] Pang Z., Chong J., Zhou G., de Lima Morais D.A., Chang L., Barrette M., Gauthier C., Jacques P.-É., Li S., Xia J. (2021). MetaboAnalyst 5.0: Narrowing the gap between raw spectra and functional insights. Nucleic Acids Res..

[70] Shannon P., Markie A., Ozier O., Baliga N.S., Wang J.T., Ramage D., Amin N., Schwikowski B., Ideker T. (2003). Cytoscape: A Software Environment for Integrated Models of Biomolecular Interaction Networks. Genome Res..

